# Description of adverse events among adult men following voluntary medical male circumcision: Findings from a circumcision programme in two provinces of South Africa

**DOI:** 10.1371/journal.pone.0253960

**Published:** 2021-08-17

**Authors:** Evans Muchiri, Salome Charalambous, Sibuse Ginindza, Mpho Maraisane, Tintswalo Maringa, Peter Vranken, Dayanund Loykissoonlal, Vincent Muturi-Kioi, Candice M. Chetty-Makkan

**Affiliations:** 1 The Aurum Institute, Johannesburg, South Africa; 2 School of Public Health, University of the Witwatersrand, Johannesburg, South Africa; 3 CDC South Africa, Pretoria, South Africa; 4 National Department of Health, Johannesburg, South Africa; 5 International AIDS Vaccine Initiative (IAVI), Nairobi, Kenya; Weill Cornell Medical College, UNITED STATES

## Abstract

**Background:**

Clinical trials showed strong evidence that voluntary medical male circumcision (VMMC) reduces the acquisition of HIV among heterosexual men by up to 60%. However, VMMC uptake in East and Southern Africa remains suboptimal, with safety concerns identified as a barrier to uptake. We investigated the occurrence and severity of adverse events (AEs) in a routine VMMC programme implemented in Gauteng and North West provinces of South Africa.

**Methods:**

We describe the frequency and characteristics of AEs using routinely collected data from a VMMC programme implemented between 01 May 2013 and 31 December 2014. The surgical procedure was provided at fixed clinics and mobile units in three districts. Adult men undertaking the procedure were referred for follow-up appointments where AEs were monitored.

**Results:**

A total of 7,963 adult men were offered the VMMC service with 7,864 (98.8%) met the age and consent requirements for inclusion in a research follow-up after the surgical procedure and were followed-up for potential AEs. In total, 37 (0.5%) patients reported AEs post-surgery with infection [11 (29.7%)] and excessive bleeding [11 (29.7%)] commonly reported AEs. In terms of severity, 14 (37.8%) were classified as mild, 13 (35.1%) as moderate, and 10 (27.0%) as severe. Further, 32 (86.5%) of the AEs were classified as definitely related to the surgical procedure, with 36 (97.5%) of all AEs resolving without sequelae.

**Conclusion:**

The VMMC programme was able to reach adult men at high risk of HIV acquisition. Reported AEs in the programme were minimal, with the observed safety profile comparable to clinical trial settings, suggesting that VMMC can be safely administered in a programmatic setting.

## Introduction

Models on the impact of voluntary medical male circumcision (VMMC) on the HIV epidemic have reported potential for a significant reduction in HIV incidence in high burden settings of sub-Saharan Africa [1–4]. Globally, the prevalence of male circumcision was estimated at 38.7% in 2015, with majority of circumcisions carried out as part of religious and cultural practices [5].

Results from three clinical trials in Uganda, Kenya, and South Africa showed that VMMC reduced acquisition of heterosexually transmitted HIV in men by up to 60% over 24 months of follow-up [6–9]. VMMC has been recommended for use in combination with other effective strategies for a comprehensive HIV prevention package [9]. The Joint United Nations Programme on HIV/AIDS (UNAIDS) recommended increasing VMMC uptake as part of a fast track strategy to reduce HIV transmission in high prevalence countries that report low male circumcision prevalence [10,11].

Despite the proven benefits of VMMC, uptake has not been optimal [12,13]. The prevalence of traditional male circumcision varies greatly among countries in East and Southern Africa, from 20% in Uganda to more than 80% in Kenya [14]. In South Africa, the prevalence of all male circumcision is estimated at 44.7%, which is below UNAIDS/WHO target of 90% for 10–29 year olds by 2021 [15,16]. Even though VMMC is generally accepted among traditionally non-circumcising communities, programmes in sub-Saharan Africa continue to face challenges with demand creation, and high uncertainty around why eligible men prefer not to take up VMMC [13,17].

Reported barriers to VMMC uptake include: limited access to the service; pain caused by the procedure; cultural influences; receiving the VMMC service from a female caregiver; and the cost of the procedure [16,18–20]. Evidence from a systematic review investigating barriers and facilitators of VMMC in countries with low VMMC prevalence and high HIV burden found that the top three barriers were perceptions of the procedure as a foreign cultural practice, fear of pain, and the perception that circumcision was not necessary [15,20,21]. While facilitators included benefits such as reducing health risks and improved hygiene, family and peer support of VMMC, and enhanced sexual performance and satisfaction [20,21].

A serious concern is whether VMMC can be provided safely to large numbers of adult men in developing countries [22–24]. Adverse events (AEs) related to circumcision vary significantly; reported to be high among men circumcising in traditional (non-clinical) settings at 35.2% and among men circumcising in clinical settings in Kenya at 17.7% with bleeding and wound infection commonly reported [22]. Timing of adverse events among men undertaking VMMC has been described to differ by country and method of circumcision with AEs reporting hampered by participants not attending all follow-up visits as recommended. Three randomised controlled trials of VMMC in Kenya, South Africa, and Uganda reported adverse events of 1.7%, 3.6%, and 7.6% respectively, with the majority classified as mild or moderately severe, and noted to resolve with treatment [6–8]. The frequency of adverse events is a widely used indicator of VMMC programme quality with studies suggesting that addressing safety issues related to VMMC may improve uptake of the service [25].

The Republic of South Africa set targets related to scaling up the practice of VMMC and incorporated this into the National Strategic Plan, with an aim to reach 80% of men aged 15–49 years with circumcision services [3,26]. As a result, VMMC clinics opened up mainly targeting high HIV burden districts in South Africa to ensure that the service was readily available to communities [26]. However, the uptake of VMMC in South Africa has not been optimal even with availability of circumcision procedures that minimize discomfort and recovery time. There is limited published literature in South Africa on adverse events among men undergoing VMMC in a programme setting, outside controlled clinical trials, to inform the safety of scaling up the programmes [25].

We investigate the occurrence and severity of adverse events reported in a VMMC programme implemented in North West and Gauteng provinces of South Africa between 01 May 2013 and 31 December 2014. These findings will inform the safety profile of the procedure among adult men undertaking VMMC in a programmatic setting.

## Methods

We retrospectively analysed data that were collected from a routine VMMC programme implemented by The Aurum Institute between 01 May 2013 and 31 December 2014 among clinics in three districts, Ekurhuleni North (EKN), Ngaka Modiri Molema (NMM) in Gauteng Province, and Dr Ruth Segomotsi Mompati (RSM) in North West Province. The VMMC services were provided to men 10 years and above at fixed clinics and mobile units using qualified clinicians where the majority of eligible clients were offered the forceps-guided medical circumcision and a small minority offered dorsal slit method as per WHO recommendations [27,28]. Qualified clinicians including medical doctors, clinical associates, and registered nurses completed a one-day theoretical course, followed by a four-day practical training during which the clinician performed at least twenty procedures under observation from a certified trainer approved by the National Department of Health (NDoH) of South Africa. The training also included client follow-up and adverse event reporting and management. Standard screening procedures were conducted, where any patient with existing medical comorbidities were first managed before the surgical procedure was performed. Circumcised men were either asked to come back to the VMMC clinic (in EKN) or were referred to an alternative clinic (in NMM and RSM) for follow-up appointments 2 days and 7 days after the surgical procedure. For this study, we only analysed data from adult participants aged 18 years and above, who had provided written informed consent for the research component of the programme, underwent surgery, and had complete data on AEs.

Demographic, social and clinical information, and sexual risk behaviour were collected during the screening interview and directly entered into a secure laptop on-site at the clinic. A unique VMMC medical reference number was assigned to each participant at each data collection point. All data were stored in a secure server managed by the Aurum Institute.

Participants were taken through a pre-circumcision counselling session on the need to abstain from any sexual activity for the 6-week period after the procedure to allow for adequate healing. The counselling session further explored care during the healing process, the effect of VMMC on HIV infection risk, behaviour that could negate the benefits of VMMC, VMMC benefits to sex partners, and the need for condom use while circumcised. We judged participants’ knowledge of the healing process to be comprehensive if they correctly answered all seven questions administered after the pre-surgery counselling. Participants were also asked about their employment status in the previous three months, and steady employment was used to describe those who had a consistent source of income including permanent employment.

Patients were instructed to contact our trained nurses if any unexpected symptoms appeared at any time throughout the recovery period. Patients were expected to return to the appointed clinics for scheduled visits (2 days and 7 days after the procedure) and telephonic follow-up calls were made when a patient missed any scheduled clinic visit. Adult men experiencing AEs after medical circumcision were assessed to determine if these were related to sexual activity during the healing period. The AEs were identified during follow-up telephone interviews initiated by the participants to a clinical staff based at the study site or an assessment post-surgery conducted in person at the site during one of the scheduled visits. Clinicians and research assistants were further asked to probe to minimize concealing or exaggerating resumption of sexual activity. The severity of the AEs observed post-surgery used the standard clinical grading of mild, moderate, and severe with a determination of whether the AEs were related to the surgery determined as per WHO guidelines on severity and timing [29]. Mild classification indicated minimal or no intervention was required beyond reassurance and observation, while moderate classification included AEs that were neither mild nor severe, required intervention, and were usually managed on-site. Severe classification required extensive intervention with hospital referral or specialist input. The relatedness of AEs to the VMMC procedure used WHO guidelines to categorize these into definite related, likely/possibly related, likely unrelated, and definitely unrelated [29]. Infection of the circumcision wound as a result of applying a traditional remedy, or displacement of a device due to sexual activity, were considered definitely related to the procedure, as neither would have occurred had there not been VMMC. Regardless of relatedness, all AEs were recorded and reported, even when the AE seems to be completely unrelated.

We analysed data using STATA version 15 (StataCorp, Texas, USA). We summarized socio-demographic characteristics using means and standard deviations for continuous variables and frequencies and associated proportions for categorical variables. The outcome indicator of adverse events was summarised using frequencies and proportions, and bivariate associations with baseline characteristics determined as statistically significant if p≤ 0.05. We did not conduct any inferential analyses, as the number of observed adverse events were few, and with insufficient variation for risk factor analyses.

The VMMC study had ethics approval obtained from the Human Research Ethics Committee of University of the Witwatersrand (150602). This project was also reviewed and cleared in accordance with the US Centers for Disease Control and Prevention (CDC) human research protection procedures and was determined to be non-research, program evaluation.

## Results

### Sample characteristics and response rates

**Fig 1:**
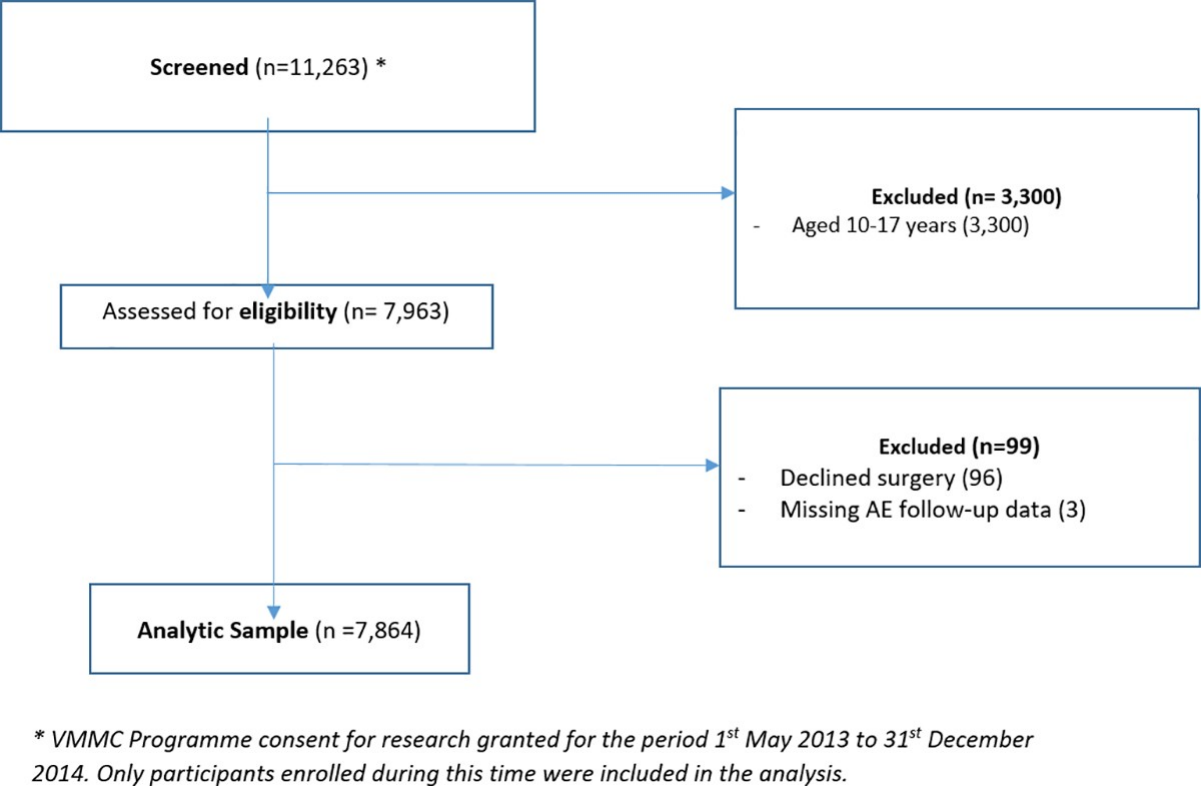
Flow of participants through the voluntary medical male circumcision (VMMC) programme among adult men in Gauteng and North West provinces of South Africa between 01 May 2013 and 31 December 2014.

Fig 1 describes the flow of participants during the programme recruitment. Of the 7,963 participants offered the VMMC service, 7,864 met the inclusion criteria and were included in the study. Follow-up data on adverse events were also available for these participants and were included into the final analytical set.

A summary of socio-demographic characteristics appears in Table 1. Of the 7,864 participants, 3,455 (43.9%) were aged between 18 and 24 years, while 7,136 (90.7%) participants had never married. In addition, 4,747 (60.4%) participants had no form of employment, and 7,722 (98.2%) had comprehensive knowledge on benefits and implications of VMMC as assessed during the pre-circumcision counselling. Majority of the participants, 5,581 (71.0%), were Nguni language speakers (iSiZulu/isiXhosa/Sepedi/Tsonga). In conducting the circumcision, the majority of the surgical procedures used the forceps method of foreskin removal with 7,616 (96.8%), while 161 (2%) of the surgical procedures used the dorsal slit method (See Table 1).

**Table 1:** Baseline characteristics of adult men presenting for a circumcision programme implemented in Gauteng and North West provinces of South Africa (2013–2014).

Characteristics	n = 7,864	(%)
Age years		
18–24	3,455	(43.9)
25–34	3,138	(39.9)
35–44	958	(12.2)
45+	313	(4.0)
Language		
Sesotho/Setswana	922	(11.7)
Nguni (iSiZulu/isiXhosa/Sepedi/Tsonga)	5,581	(71.0)
English/Afrikaans	211	(2.7)
Other	1,019	(13.0)
Missing	131	(1.7)
Marital Status		
Never Married	7,136	(90.7)
Married	641	(8.2)
Separated/Divorced/Widow	37	(0.5)
Missing	50	(0.6)
Employment Status		
None	4,747	(60.4)
Some	494	(6.3)
Steady	2,569	(32.7)
Missing	54	(0.7)
HIV status		
Negative	5,892	(74.9)
Positive	239	(3.0)
Not done	1,547	(19.7)
Missing	186	(2.4)
Condom use		
Never	2,550	(32.4)
Inconsistent–some/most times	2,744	(34.9)
Consistent–every time	1,989	(25.3)
Missing	581	(7.4)
Sexual partners		
None	1,454	(18.5)
One	3,904	(49.6)
Two or more	2,418	(30.8)
Missing	88	(1.1)
Circumcision method		
Forceps	7,616	(96.8)
Dorsal Slit	161	(2.1)
Sleeve	2	(<0.1)
Other	44	(0.6)
Missing	41	(0.5)
VMMC Knowledge scores from 7 statements		
Limited (0–6)	10	(0.1)
Comprehensive (7)	7,722	(98.2)
Missing	132	(1.7)

Reported adverse events are presented in Table 2. In total, 37 (0.5%) adult men of the 7,846 participants experienced AEs after surgery and were included in the analyses. Of the 37 AEs after the VMMC surgical procedure, 2 (5.0%) were reported the same day of surgery, 13 (35.1%) were reported between –1–2 days post-surgery, 17 (45.9%) reported between 3–7 days post-surgery, and 5 (13.5) reported after 7 days of surgery. The severity grading of the AEs reported indicated that 14 (37.8%) were mild, 13 (35.1%) moderate, and 10 (27.0%) severe. Infection and excessive bleeding were the most common AEs reported at 11 (29.7%) each, while wound disruption during the healing period represented 6 (16.2%) of all AEs. The 10 AEs deemed to be severe were observed as follows: 2 as wound disruption, 3 as excessive bleeding, 1 as an infection or sepsis, 1 had insufficient skin removed, 1 was a swelling or hematoma, and 2 were other adverse events. The assessment of the adverse event indicated that 32 (86.5%) of the AEs were definitely related to the circumcision procedure and 36 (97.3%) of the observed AEs were reported to have resolved without sequelae, while the outcome of one was unknown. In relation to the circumcision procedure, 36 (97.3%) of AEs observed were from the forceps procedure, while 1 (2.7%) emanated from the dorsal slit procedure. However, the majority of procedures were performed using the forceps procedures, as noted above.

**Table 2:** Description of adverse events (AEs) reported post-surgery in adult men in North West and Gauteng provinces of South Africa (May 2013 –December 2014) (n = 7,864).

Characteristics	n = 37	(%)
Timing of AEs reported		
Day of surgery	2	(5.4)
Between 1 and 2 days of surgery	13	(35.1)
Between 3 and 7 days of surgery	17	(45.9)
After 7 days of surgery	5	(13.5)
Severity		
Mild	14	(37.8)
Moderate	13	(35.1)
Severe	10	(27.0)
Type		
Appearance/Wound disruption–Post	6	(16.2)
Excessive bleeding	11	(29.7)
Infection/Sepsis	11	(29.7)
Insufficient skin removed	4	(10.8)
Other adverse event	2	(5.4)
Swelling/Hematoma	3	(8.1)
Related to circumcision		
Definitely related	32	(86.5)
Possibly related	1	(2.7)
Likely related	1	(2.7)
Definitely Unrelated	3	(8.1)
Adverse Event Resolution		
Resolved with no permanent damage	36	(97.3)
Unknown	1	(2.7)

Results from Table 3 report bivariate associations between baseline factors and reported adverse events post-surgery. Results indicate no statistically significant risk factors for developing AEs for all risk factors assessed at the p-value <0.05 significance level.

**Table 3:** Bivariate associations between adverse events (AEs) and socio-demographic characteristics (n = 7,864) of adult men in Gauteng and North West provinces of South Africa (01-May-2013 and 31-December-2014).

	No AEs		AEs observed		Fisher’s exact p-value
Characteristics	n = 7,827	(%)	n = 37	(%)	
**Age categories (years)**					(0.45)
18–24	3,443	(99.7)	12	(0.3)	
25–34	3,119	(99.4)	19	(0.6)	
35–44	953	(99.5)	5	(0.5)	
45+	312	(99.7)	1	(0.3)	
**Marital Status**					(0.35)
Never Married	7,104	(99.5)	32	(0.5)	
Married	636	(99.2)	5	(0.8)	
Separated/Divorce	20	(100.0)	0	(0.0)	
Widow	17	(100.0)	0	(0.0)	
**Employment status**					(0.60)
None	4,727	(99.6)	20	(0.4)	
Some	492	(99.6)	2	(0.4)	
Steady	2,554	(99.4)	15	(0.6)	
**Language**					(0.15)
Sesotho/Setswana	920	(99.8)	2	(0.2)	
Nguni (iSiZulu/isiXhosa/Sepedi/Tsonga)	5,557	(99.6)	24	(0.4)	
English/Afrikaans	209	(99.0)	2	(1.0)	
Other	1,011	(99.2)	8	(0.8)	
**Circumcision method**					(0.63)
Forceps	7,580	(99.5)	36	(0.5)	
Dorsal slit	160	(99.4)	1	(0.6)	
Sleeve	2	(100.0)	0	(0.0)	
Other	44	(100.0)	0	(0.0)	
**HIV Status**					(0.10)
Negative	5,867	(99.6)	25	(0.4)	
Positive	236	(98.7)	3	(1.3)	
**Sexual partners**					(0.18)
None	1,451	(99.8)	3	(0.2)	
One	3,886	(99.5)	18	(0.5)	
Two or more	2,403	(99.4)	15	(0.6)	

## Discussion

Even with compelling evidence that VMMC reduces HIV acquisition in men, uptake has been sub-optimal in East and Southern Africa. Safety concerns around the VMMC service have the potential to deter service uptake [13,30–32]. The VMMC programme among adult men in Gauteng and North West Provinces of South Africa was able to reach both mature men aged 25–45 years, and married men.

This study sought to assess the frequency and characteristics of adverse events following a VMMC programme. The study found that the incidence of reported adverse events following VMMC was minimal in the programme, with observed rates comparable or lower to those observed in clinical trials evaluating VMMC effectiveness [6–8]. In a multi-site non-randomised, prospective cohort study evaluating the safety of PrePex device for male circumcision in six clinics across South Africa, 2.5% of PrePex MMC device participants experienced adverse events [33]. In a prospective study comparing traditional non-clinical and clinical circumcision procedures in Bungoma, Kenya, adverse event rates were observed to be higher [22]. However, these clinical trials had much longer period of follow-up period monitoring for AEs incidence, unlike in this study where follow-up was not as active after 7 days post-surgery.

The incidence of adverse events reported in this VMMC programme was low in all age groups and did not differ significantly by marital or employment status. Reported AEs from clinical trials and the VMMC programme data may not be strictly comparable, as clinical trials have more active follow-up procedures that may lack in a routine programme [34]. However, the low incidence of adverse events reported in this study suggests that the quality of the programme is good and the findings are reassuring for broader implementation efforts.

Findings emanating from this study did not identify baseline factors that were significantly related to AEs among adult men. This was as because very few AEs were observed in the programme. The nature of some of the AEs observed suggested may suggest earlier resumption of sexual activity than recommended in the instructions following assessment and probes by clinicians and research staff. Studies have reported that a significant proportion of adult men taking part in VMMC programmes resume sex earlier than the 42-day period recommended by WHO [35]. Resuming sexual activity early, before the surgical wound has completely healed, may negate VMMC benefits in HIV prevention [36] and has been showed to increase the risk of HIV transmission to female partners of HIV-infected men [37].

These analyses had potential limitations. Generally, all AEs may have been underreported as the patient follow-up in our VMMC programme was not as active as it would have been in a clinical trial, with potential for underreporting of AEs in this real-life implementation programme. A further limitation is the potential underreporting of AEs experienced due to cultural reasons [38]. In some African cultures, men are less likely to seek hospitalisation if this may portray them as weak, thus the potential for underreporting of adverse events [39,40]. Attempts to mitigate potential beliefs on AEs reporting were addressed during the pre-surgery counselling to clients by stressing the need for prompt intervention at a health facility if any adverse event was experienced. Based on the knowledge assessment scores, the pre-surgery counselling was well understood by the clients, and we believe participants were empowered to make safer decisions and seek care if indicated.

Clinicians involved in performing the circumcision procedures in the VMMC programme were extensively trained by institutions approved by both the Department of Health in South Africa and PEPFAR. This may not be feasible in low resource settings where lower cadres of clinical staff may be required to perform this procedure. Even with extensive training of clinical staff involved in this study, there is potential for differences in how AEs were defined and rated due to inter-rater variability, hence potential source for bias. Evidence from a study assessing the quality of VMMC service offered by mid-level health workers in South Africa showed no difference in the number of adverse events reported when comparing clinical associates and medical doctors [41]. Further, this study reported on AEs from a VMMC programme where majority of procedures were performed using the Forceps guide method, it is not clear how this would differ in setting where other methods are preferred. We were not able to conduct more elaborate risk factor analysis using models to identify factors associated with AEs following VMMC because of low number of observed AEs. Additional data are needed from longer time frames or multiple sites to further understand risk factors. The project was implemented as part of a routine service as such data collected were not as detailed as would be required for a clinical trial. Some indicators including employment status were not as specific and we could not tell variation of AEs by type of employment as potential differences by type of labour would be expected.

## Conclusion

This study found very low incidence of AEs reported among adult men above 18 years following the circumcision procedures performed in a VMMC programme in South Africa. The low incidence of observed AEs in this programmatic setting suggest that scaling up VMMC programmes can be done in a safely manner without an apparent increase in reported AEs.

## Supporting information

S1 File (XLS)
